# Gypsum-Based Composites with Recycled PP/HDPE Pellets for Circular Material Development: A Comprehensive Characterisation

**DOI:** 10.3390/ma18174037

**Published:** 2025-08-28

**Authors:** Daniel Ferrández, Alicia Zaragoza-Benzal, Pedro Carballosa, José Luis García Calvo, Paulo Santos

**Affiliations:** 1 Escuela Técnica Superior de Edificación, Universidad Politécnica de Madrid, Avda. Juan de Herrera, 6, 28040 Madrid, Spain; daniel.fvega@upm.es (D.F.); alicia.zaragoza@upm.es (A.Z.-B.); 2 Instituto de Ciencias de la Construcción “Eduardo Torroja” (IETcc-CSIC), C/Serrano Galvache 4, 28033 Madrid, Spain; carballosa@ietcc.csic.es (P.C.); jolgac@ietcc.csic.es (J.L.G.C.); 3 University of Coimbra, Department of Civil Engineering, ISISE, ARISE, 3030-788 Coimbra, Portugal

**Keywords:** recycled PP/HDPE pellets, gypsum-based composites, circular economy, physico-mechanical characterization, life cycle assessment

## Abstract

Managing plastic waste is a great challenge for today’s society, and it is increasingly necessary to find solutions to the large amount of plastic waste dumped annually in the oceans. The main objective of this research is to perform a comprehensive characterisation of different gypsum-based materials incorporating recycled PP/HDPE pellets from the recycling of discarded fishing nets in the Mediterranean Sea. For this purpose, composites were developed with a partial substitution of the original material by these pellets, up to 30% by volume, while maintaining a water/gypsum ratio of 0.65 by mass. The results showed that even in the most unfavourable case, with a 30% replacement in volume by these recycled pellets, flexural (2.72 MPa) and compressive (7.15 MPa) strengths higher than those required by the standards were obtained, with good integration of the residue in the matrix. Also, there was a decrease in total water absorption of up to 20.5% compared to traditional gypsum. The thermal behaviour study showed that a minimum conductivity value of 292.3 mW/m K was obtained, implying a decrease of 14.9% from the control series. In addition, a life cycle analysis was conducted, obtaining a reduction in environmental impact of up to 13.1% in terms of CO_2_ equivalent emissions. Overall, the composites obtained represent a sustainable alternative to producing prefabricated plates and panels for building construction.

## 1. Introduction

The development of plastic products has increased in recent decades and has brought many advantages to everyday life [1]. However, the management of plastics remains a significant challenge, and there is a growing need to find solutions to mitigate this global problem [2]. Recent studies indicate that plastic consumption, far from being solved, will increase to 1.2 billion tons by 2060 [3]. Considering that these products take approximately 500 years to decompose in landfills and their average lifespan is 10 years [4], finding new ways to recycle and reuse this waste is urgent. This is why the European Commission has identified plastic waste as a priority in its action plan for moving towards a circular economy [5]. Furthermore, the European Green Pact highlights that the construction sector is a key player in this process and needs to move towards responsible use of resources in this industry [6].

Consequently, the use of plastic waste as a secondary raw material in the development of construction materials has gained increasing attention due to society’s greater environmental awareness and the need to develop new sustainable solutions under circular economy criteria [7]. This work focuses on developing gypsum-based composites that incorporate recycled plastic pellets and on characterising and studying their potential applications. In recent decades, gypsum materials have generated increasing interest in the development of prefabricated and modular construction elements [8]. This is because these materials present excellent hygrothermal regulation properties [9], great versatility for forming low-cost, easy-to-handle prefabricated boards and panels [10], and a wide range of non-structural applications that allow for the integration of secondary raw materials into their matrix [11]. For this reason, it is not surprising that numerous researchers have focused their studies on integrating recycled plastic pellets into gypsum composites. In Table 1, the current state of knowledge related to this research is analysed through a Web of Science search. For this purpose, the last five years (2020–2025) and the existing literature in the English language have been taken into consideration using the following keywords: ((“Gypsum” OR “Plaster”) AND (“Pellet” OR “Aggregate”) AND (“Plastic” OR “Polyethylene” OR “Polypropylene” OR “Polyurethane” OR “PET” OR “PVC” OR “EPS” OR “XPS”) NOT (“Cement” OR “Geopolymer” OR “FIBRE*” OR “FIBER*”)).

In general terms, several conclusions about using recycled plastic pellets in gypsum-based materials can be drawn from the literature review in Table 1. Firstly, a decrease in mechanical properties (flexural and compressive strength) is observed due to weak bonding in the gypsum-pellet interphase, leading to preferential breakage points [13,14,15,19]. Likewise, a decrease in the bulk density and thermal conductivity of gypsum composites is observed with the incorporation of these secondary raw materials. However, this reduction depends on the nature of the added plastic residue [12,15,17,18]. On the other hand, an improvement in the water resistance of these gypsum-based materials has also been observed due to the hydrophobic nature of the plastic residues [13,17]. In some cases, an increase in the internal porosity of the composites was observed [19]. Finally, most studies highlight the benefit of incorporating recycled raw materials to improve the circularity of construction products and reduce the environmental impact associated with the production of construction materials [16,17,19].

This research focuses on the innovative application of polypropylene/high-density polyethylene (PP/HDPE) pellets obtained from recycling marine rope recovered from the Mediterranean Sea for use in the construction industry. The motivation arises from alarming figures that estimate the amount of plastic waste dumped annually into the oceans at more than 8 million tonnes [20], which in turn contributes to the formation of microplastics that aggravate marine pollution and damage ecosystems [21,22]. Sub-target 14.1 of the Sustainable Development Goals (SDGs) highlights the need to reduce the dumping of marine litter, especially plastic waste [23]. Its amount has increased by 42% in the last 40 years [24]. The Mediterranean Sea in Europe, due to its semi-enclosed nature, high population and tourist activity along its coasts, and high marine circulation, accumulates a large concentration of floating plastics that need to be managed and given a second life [25].

According to recent studies, 5.7% of fishing nets are dumped into marine ecosystems [26]. Their non-biodegradable nature makes the decomposition process extremely slow (about 600 years), turning them into harmful waste with long-term negative consequences for the environment [27]. However, this high durability has led to these waste fibres being used as a reinforcement material in mortars and concretes [28]. In particular, nylon fibres have been added at a rate of 1–2% by mass to improve the mechanical strength of these materials and increase their ductility [29]. However, without proper treatment, these fibres contain a high chloride content that compromises the durability of cementitious materials, mainly reinforced ones [28,29]. For this reason, in recent years, Romero-Gómez et al. have carried out several studies applying these fibres to the development of gypsum composites, improving their mechanical strength [30] and resistance to water and high temperatures [31], while obtaining beneficial effects not only from a technical point of view, but also from an environmental point of view when using them in the production of prefabricated products [32]. However, no studies have been found to date that transform this marine plastic waste into pellets and use this granular material as an admixture in gypsum composites.

Therefore, the main objective of this research is to conduct a comprehensive characterisation of novel gypsum-based materials incorporating recycled PP/HDPE pellets. For this purpose, a progressive substitution of the original gypsum material with these secondary raw materials of up to 30% by mass is carried out. Subsequently, a complete experimental campaign, including mechanical and hygrothermal characterisation and environmental impact analysis of these building materials, is carried out, followed by a critical discussion of the results.

## 2. Materials and Methods

This section describes the raw materials used to produce the gypsum-based composites, the sample preparation process, and the experimental programme developed.

### 2.1. Employed Raw Materials

**Figure 1:**
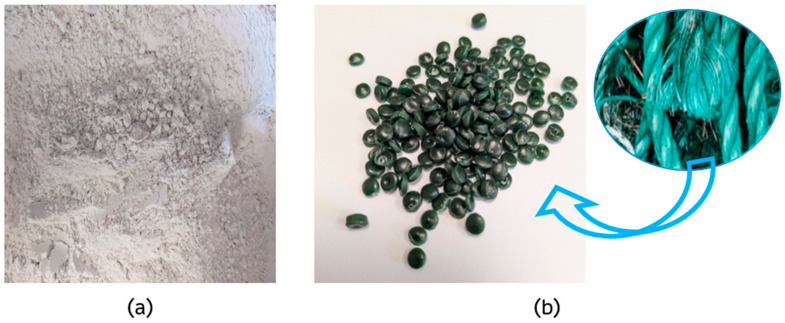
Raw materials used: (**a**) gypsum; (**b**) recycled PP/HDPE granules from fishing nets.

Construction gypsum, recycled PP/HDPE pellets, and water were used in this research. Figure 1 shows a picture of the raw materials.

***Binder***: Iberyola E-35 gypsum, supplied by Saint-Gobain Placo Ibérica (Madrid, Spain), was used as a binder material. This type of gypsum has a type A classification according to EN 13279-1:2008 [33]. Due to its high purity (>92%) and quality, it has been frequently used in various research works with gypsum-based composites [8]. Its main characteristics are shown in Table 2.

***Recycled PP/HDPE pellets***: obtained from high-density polyethylene and polypropylene marine ropes recovered from the Mediterranean coasts, which were recycled to give them a second useful life. The mass distribution of the plastics that make up these pellets is 54% PP and 46% HDPE. The properties of these raw materials supplied by Gravity Wave (Alicante, Spain) are listed in Table 3.

***Water***: Tap water from the Canal de Isabel II (Community of Madrid, Spain) was used to mix the gypsum-based composites. This potable water is suitable for human consumption and has been successfully used in previous research [36]. Its main characteristics are as follows [37]: pH 8.04, free chlorine 0.03 mg/L, electrical conductivity (20 °C) 157.06 µS/cm, calcium 20.3 mg/L, and hardness 55.67 mg/L [38].

#### Preparation Process and Mix Proportions Used

**Figure 2:**
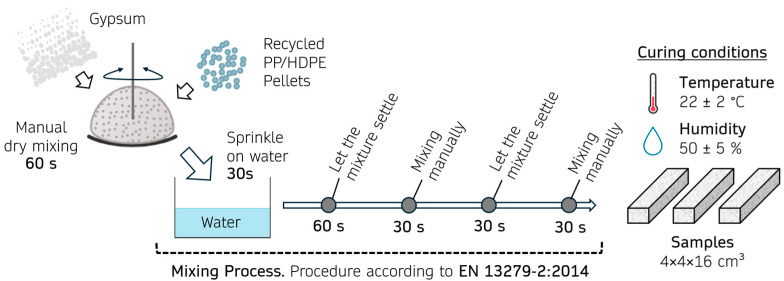
Preparation process of the gypsum-based composites incorporating recycled PP/HDPE pellets.

During sample preparation, the recommendations and methods described in EN 13279-2:2014 were followed [38]. The process used during the preparation is shown schematically in Figure 2.

The proportions used to prepare the different gypsum-based composites are shown in Table 4. Recycled PP/HDPE pellets were incorporated by partial volume substitution of the original raw material. The water/gypsum mass ratio was fixed at 0.65, which allowed for achieving plastic and workable consistency, following the shaking table method [38]. All the composites produced exhibited a diameter of 165 ± 10 mm after the shaking table test. The maximum substitution percentage was experimentally established at 30% pellets, since this corresponds to the limit (156 mm diameter after the shaking table test) for achieving the required workability of the mixture without altering the previously defined water/gypsum ratio.

**Figure 3:**
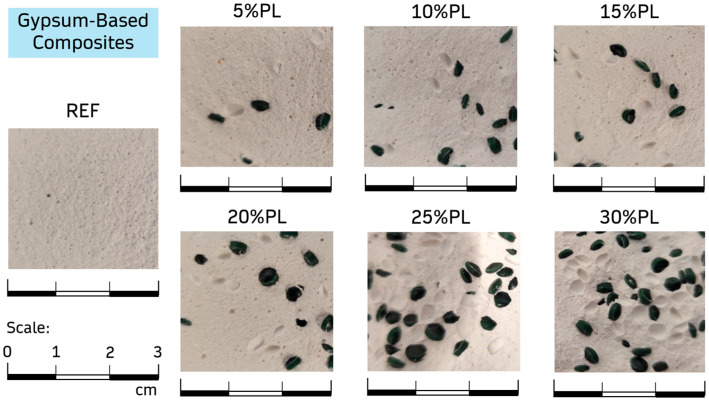
Cross-section of the different gypsum-based composites developed in this research.

Once the samples were cured under laboratory conditions and before the tests, they were dried in an oven at 40 ± 2 °C and a relative humidity of 50 ± 5% RH according to the regulations [38]. Figure 3 shows an image from the cross-section of 4 × 4 × 16 cm^3^ specimens for each sample produced. The distribution of the recycled PP/HDPE pellets inside the hardened gypsum matrix can be appreciated.

### 2.2. Experimental Programme

The experimental campaign for this research was developed in the following phases: mechanical characterisation, study of the hygrothermal properties, and analysis of the environmental impact.

#### 2.2.1. Mechanical Properties

**Figure 4:**
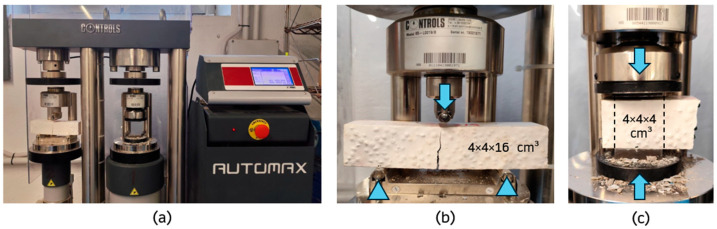
Mechanical tests: (**a**) testing equipment; (**b**) flexural test; and (**c**) compression test.

For the mechanical characterisation of the different gypsum-based composites, the following tests were carried out on 4 × 4 × 16 cm^3^ specimens:

- ***Surface hardness***: determined with the aid of a Shore C durometer Baxlo (Barcelona, Spain), according to the indications of the UNE 10242:2023 standard [39]. The measurements were obtained on two 4 × 16 cm^2^ plane-parallel faces, with five measurements per face, spaced a minimum of 20 mm apart, and three specimens from each series were tested.
- ***Flexural strength***: determined according to the EN 13279-2:2014 standard [38] (Figure 4). A three-point flexural test was performed with the aid of AUTOMAX^®^ equipment (Sacramento, CA, USA), testing a total of three specimens for each mix at a loading rate of 40 N/s.
- ***Compressive strength***: determined according to the EN 13279-2:2014 standard using six samples per mixture obtained from the flexural strength test (Figure 4). The compression module of the AUTOMAX^®^ hydraulic press (Sacramento, CA, USA) was used, with a loading rate of 400 N/s.
- ***Scanning Electron Microscopy (SEM)***: used to evaluate the microstructure of the composites produced. For this purpose, the cold cathode field emission scanning electron microscope model FE-SEM HITACHI S-4800 (Krefeld, Germany) was used, equipped with the Bruker Nano detector model X-Flash Detector 5030 (Berlin, Germany). In addition, the tested samples were pre-coated with gold foil to ensure the conductivity of the electron beam, using a Q150T Plus metalliser (Laughton, East Sussex, UK).

#### 2.2.2. Hygrothermal Properties Assessment

This section investigates the behaviour of gypsum-based composites under water action and their thermal behaviour. For this purpose, the following tests were carried out:

- ***Capillary water absorption***: calculated using the procedure adapted from EN 1925:1999 for natural stone [40]. It represents the water mass that each composite can absorb per unit time and surface area. Three samples were taken from each series with dimensions of 4 × 4 × 16 cm^3^, and the following equation was used:
  (1) Coefcap=Wt−W0As·t·100 kg/m2·s12
  where $W_{t}$ is the mass (kg) of the specimen at time t = 1, 3, 5, 10, 15, 20, 40 min; $W_{0}$ is the mass of the dried specimen (kg); $A_{s}$ is the area of the submerged face, which is 4 × 4 cm^2^; and $\sqrt{t}$ is the square root of the time (s12). The specimens are placed vertically, separated from the bottom by a grid that allows water to pass through, and submerged to a depth of 5 ± 1 mm during the test.
- ***Total water absorption***: it refers to the total water mass that the composites can absorb when immersed in water. It is determined according to EN 520:2004 [41] on specimens measuring 15 × 15 × 2 cm^3^. For this purpose, the specimens, which have been previously dried in an oven at 40 ± 2 °C for 24 h and weighed, are immersed at room temperature in a container of water, separated from the bottom with the help of a grid. After 120 ± 2 min, the samples are removed, the excess surface water is removed, and they are weighed again. In this way, by knowing the initial and final (saturated) weight, the total water absorption coefficient is obtained as a percentage.
- ***Open porosity***: determined with the aid of a 0.01 g hydrostatic balance, according to the procedure described in EN 1936:2006 for natural stone [42]. This parameter is the percentage ratio between the open pores’ volume and the specimen’s apparent volume. It is calculated according to Equation (2) using three specimens of each 4 × 4 × 16 cm^3^ gypsum-based composite:
  (2) $${Open}_{por}=\left(\frac{W_{s}-W_{0}}{W_{s}-W_{i}}\right)\cdot 100\;\left[\%\right]\;$$
  where $W_{s}$ is the saturated mass of the specimen (g); $W_{0}$ is the mass of the dried specimen (g); and $W_{i}$ is the mass of the specimen immersed in water (g).
- ***Bulk density***: determined under the procedure set out in UNE 102042:2023 [39]. For this purpose, a total of three 4 × 4 × 16 cm^3^ samples were used. This bulk density is the quotient between the specimen’s mass and apparent volume (256 cm^3^). A laboratory balance with a pressure of 0.01 g was used to obtain the mass.
- ***Thermal conductivity***: determined by the needle method, described in depth in the research carried out by Revuelta et al. in 2021 [43]. For this purpose, needles 60 mm in length and 2.4 mm in diameter are used; they are introduced into the gypsum mixture in its fresh state and removed once the setting process has begun, thus leaving a hollow inside the composite. Subsequently, with the material hardened after seven days and conditioned in an oven for 24 h at 40 ± 2 °C, measurements were taken at room temperature (22 °C). Three measurements were taken for each composite type, and the mean value for thermal conductivity was obtained. The equipment used for data acquisition is a Decagon KD2 Pro Thermal Properties Analyzer (Pullman, WA, USA) equipped with TR-1 probes. Before testing, the holes were filled with Arctic Silver thermal paste to ensure adequate contact between the needle and the processed material.

Finally, a numerical finite element simulation was carried out to thoroughly understand the functional performance of the new gypsum-based composites developed in this research. Two lightweight steel frame (LSF) wall configurations were modelled using the 2D finite element simulation software THERM (**Version 7.8.80**): interior partition and façade wall. This simulation allows for an accurate estimation of the thermal resistance of the evaluated envelopes [44], taking into account the energy efficiency improvement resulting from designing these circular materials. In the modelled section, the steel stud, which constitutes this system’s resistant structure, was included. This is a conflicting point, as it significantly increases heat transfer, thereby reducing thermal resistance.

#### 2.2.3. Environmental Impact Analysis

The environmental impact of the composites developed in this research was assessed following the life cycle analysis (LCA) methodology according to ISO 14040 standards [45]. LCA is a widely accepted method for evaluating the environmental performance of materials and construction systems [46,47]. This analysis aims to determine the environmental impacts of incorporating recycled PP/HDPE pellets to partially replace gypsum in the production of prefabricated elements for use in buildings. The production has been considered in a factory where gypsum and gypsum-based prefabricated elements are produced.

The life cycle stages considered were A1-A2-A3 (cradle-to-gate). For this purpose, 1 m^2^ of prefabricated panel made with REF and 30%PL composites for constructing the interior wall was established as the declared unit. Table 5 shows the inventory data: raw materials and their transport to the production plant. The energy required to produce the panels was obtained from recent literature [32], considering the following stages and electrical consumption: mixer (0.80 kWh/t), board line (2.78 kWh/t), dryer (1.11 kWh/t), and packaging (0.02 kWh/t).

**Figure 5:**
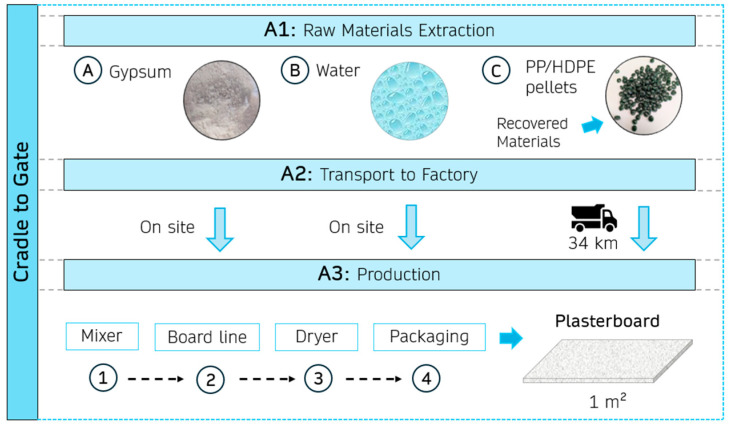
System boundary employed in the LCA of the gypsum composite studied.

The Environmental Product Declarations (EPD) for the raw materials (gypsum and recycled PP/HDPE pellets) from Spanish and European companies were used to prepare the life cycle inventory. The data available in the Ecoinvent report V3.10 database were also used for water and the energy mix in the specific case of Spain. The distances considered for transport were based on real industrial plants in the Community of Madrid (Spain). For the transport of raw materials, a freight lorry (16–32 t) was considered, taking into account only the supply of recycled PP/HDPE pellets, since tap water is supplied directly at the factory and the gypsum used is manufactured in the plant. The potential environmental impacts of the products studied were obtained using a life cycle inventory analysis (LCIA) with the CML-IA baseline v3.10 methodology. The boundary conditions of this analysis are shown schematically in Figure 5 below.

## 3. Results and Discussion

This section presents the results obtained for this research, as well as a discussion of the results.

### 3.1. Mechanical Properties

**Figure 6:**
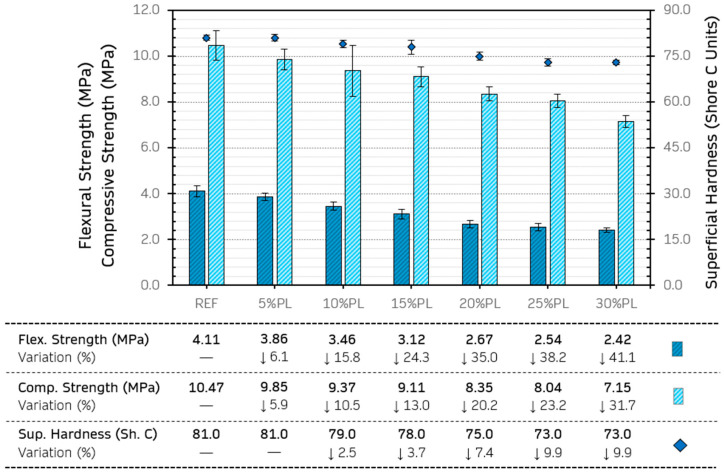
Results of the analysed mechanical properties.

Gypsum composites present an excellent opportunity to reintroduce recycled plastics into the value chain of these products in a sustainable and economically viable way [48]. However, current regulations require specific minimum mechanical strength values to consider these construction materials viable for building use. Figure 6 shows the results obtained for the mechanical strength tests of the gypsum-based composites produced.

Figure 6 shows the same trend in the three mechanical properties analysed, where the strength decreases as the recycled PP/HDPE pellet content in the matrix increases. For flexural and compressive strengths, the maximum reduction compared to the reference gypsum material without additions was 41.1% and 31.7%, respectively, in the 30%PL composite. This adverse effect on flexural and compressive strengths has been previously observed by other authors and is linked to several factors. Vidales-Barriguete et al. observed how incorporating plastic pellets resulted in higher porosity and density reduction in gypsum composites, which implied a decrease in the material’s capacity to withstand compressive and flexural stresses [49].

On the other hand, plastic aggregates do not interact chemically with gypsum and present difficulties in integrating into its crystalline network [50] (as can be seen in Section 3.1.1 of this document). This reduced integration depends mainly on the particle size of the plastic residue added. In this context, Pedreño-Rojas et al. identified weak adhesion at the gypsum–plastic interface that broke the continuity of the composite matrix and generated preferential breakage points [51]. Finally, another possible cause is associated with the modification of the dihydrate crystal formation pattern, which alters the microstructure of the composite and weakens the material [52]. In any case, all the composites produced exceeded the minimum flexural (1 MPa) and compressive (2 MPa) strength values recommended by EN 13279-2:2014 [38].

The decrease was less significant for surface hardness, reaching a maximum variation of 9.9% in the 30%PL composite compared to the reference. Romero-Gómez et al. [13] and Álvarez et al. [19] observed similar behaviour in their studies with polypropylene plastic pellets from recycled coffee pods and high-density recycled polyethylene, respectively. However, it is important to highlight the observation made by Martínez-Arredondo et al., who note in their study that the Shore scale is non-linear and that small differences between composites can involve relatively significant variations in practice [15].

**Figure 7:**
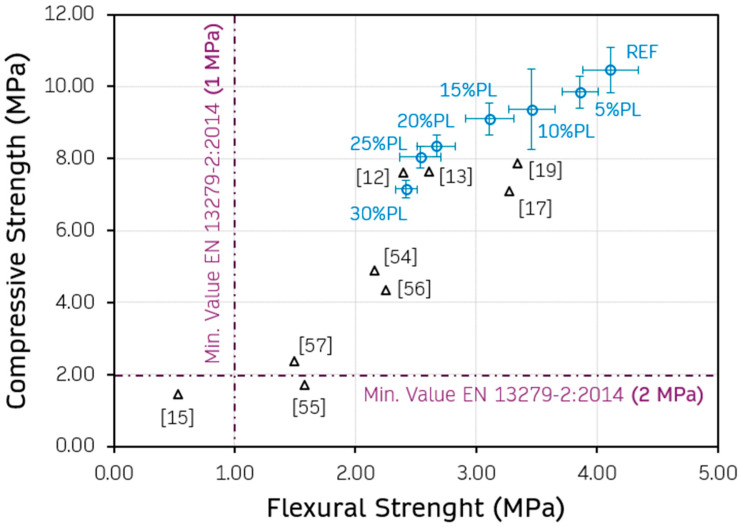
Quantitative discussion of the results: [12] plaster with a 40% wt. addition of waste CDs (PC) smaller than 4 mm; [13] plaster with 10% wt. substitution of PP smaller than 4 mm; [17] plaster with 7.5% wt. substitution of shredded LDPE (0.125–1.000 mm) from recycled bags; [19] plaster with 10% wt. substitution of recycled HDPE with a diameter of 1.4 mm; [53] gypsum with 20% wt. substitution of shredded tennis ball waste smaller than 5 mm; [54] gypsum with 4% wt. substitution of XPS smaller than 4 mm in diameter; [55] gypsum with 30% wt. substitution of shredded tyre waste smaller than 0.8 mm; and [56] gypsum with 70% wt. substitution of plastic cable waste smaller than 4 mm.

To complete the discussion in this section, Figure 7 shows the values obtained for flexural and compressive strength from various studies found in the literature, which are related to the materials analysed in this research. In this way, a comparative image can be obtained with the values for gypsum-based composites with recycled PP/HDPE pellets and their relative position to the minimum flexural and compressive strength values indicated by the standards.

Given the comparison presented in Figure 7, the gypsum-based composites produced in this research showed optimum mechanical properties compared to those obtained in other studies. However, it should be noted that Figure 7 has been constructed using the results obtained for the gypsum-based composites with the highest content of recycled material in each article analysed, and the strength could also vary with modifications in the water/binder ratio. In any case, it can be observed that the gypsum composites made with recycled PP/HDPE pellets would be optimal for their application in the manufacture of false ceiling plates and prefabricated housing panels, where no structural use or high load-bearing capacity is required.

#### 3.1.1. SEM Analysis

**Figure 8:**
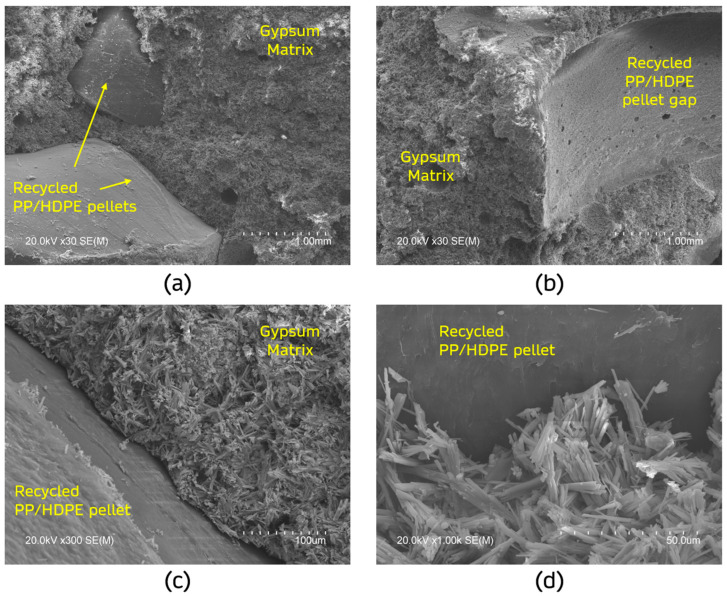
SEM analysis for sample 30%PL. Magnifications (**a**) ×30; (**b**) ×30; (**c**) ×300; (**d**) ×1000.

To better understand the microstructure of the gypsum-based composites produced, SEM analysis was carried out on the sample with the highest content of recycled PP/HDPE pellets (30%PL). The images of the inner matrix of the sample studied were taken without modifying its surface by polishing or similar techniques to preserve its original morphology and ensure maximum representativeness. These images are shown in Figure 8.

Figure 8a shows a general image of the composite matrix, where the distribution of the recycled PP/HDPE pellets within the gypsum composite can be seen. In agreement with the study by Vidales-Barriguete et al., it is observed that the gypsum matrix completely envelops the pellets during the mixing process, and no increase in porosity is observed in the composites because of the addition of plastic waste [57]. Figure 8b details the pellet detachment from the composite matrix during sample preparation. It can be seen, in line with previous studies, that the smooth surface of these pellets hinders adhesion to the gypsum matrix, so some authors have suggested the need for chemical surface treatments to correct this effect [19].

Figure 8c details the interface, visualising the transition between the added waste and the gypsum matrix. A weak bond between both raw materials is denoted, which justifies the decrease in mechanical properties as the content of recycled PP/HDPE in the samples increases [12]. Finally, Figure 8d, with higher magnification, allows for the visualisation of the characteristic acicular morphology of the gypsum crystals (CaSO_4_-2H_2_O), which evidences a correct setting of the mixture after the mixing process [58]. In the same way, the gypsum–plastic transition is observed with the formation of these dihydrate crystals on the surface of the residue, an effect observed in the research carried out by Romero-Gómez et al. [32].

### 3.2. Hygrothermal Properties

Gypsum boards are widely used as interior finishing materials in facades and as partitions between rooms in dwellings, so it is essential to know their performance regarding water absorption and thermal insulation capacity [58]. This section analyses some of the building material’s most relevant hygrothermal properties.

#### 3.2.1. Water Performance

**Figure 9:**
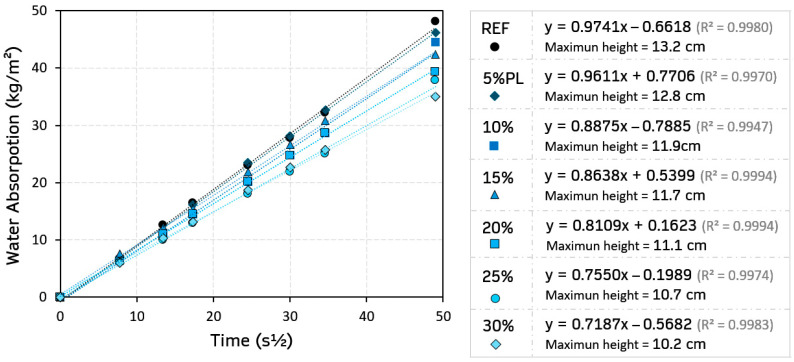
Results for the capillary water absorption test.

The performance of gypsum composites in humid environments is limited because of their high solubility and the loss of strength caused by direct contact with water [59]. For this reason, reducing capillary water absorption capacity is essential for improving the functionality of these building materials. The results obtained from this test are presented in Figure 9.

As shown in Figure 9, replacing the original gypsum material with recycled PP/HDPE pellets gradually reduced the water absorption capacity per unit area in these composites. Therefore, the greatest reduction at the end of the test was observed in the 30%PL composite, measuring 35.0 kg/m^2^-s^1/2^ (20.5% less than the traditional material, REF). This decrease in the water absorption capacity due to capillarity is also reflected in the maximum height reached by the water in this test. This phenomenon is common in this type of gypsum material with plastic waste, even if there is an increase in porosity and a relative increase in pore size [17]. Recycled PP/HDPE pellets have a hydrophobic nature, so this type of aggregate does not absorb or facilitate the passage of water through the matrix [13]. Some research has found that incorporating these plastic pellets reduces the porous surface area available for water absorption [49]. Other investigations suggest the formation of a less connected and more clogged inner porous network with the incorporation of plastic aggregates in the composite matrix, which has an impact on the decrease in the water diffusion rate inside the composite [60].

**Figure 10:**
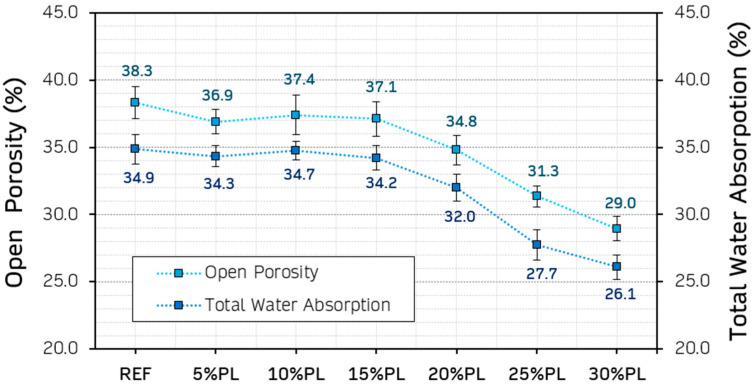
Results for open porosity and total water absorption.

The results obtained for the open porosity and total water absorption of the gypsum-based composites are shown in Figure 10. Both physical properties are of special interest in the study of these materials. The first one shows the volume of pores connected to the surface that allow the passage of liquids through the composite, meaning that a higher porosity may contribute to a decrease in durability under wet/dry cycling conditions [61]. On the other hand, total water absorption is key to the drying times of these composites, and the two properties are closely related.

A similar trend is seen for the properties in Figure 10. Both open porosity and total water absorption decreased with the addition of recycled PP/HDPE aggregates. More specifically, from 15% volume substitution onward, this change became more noticeable and significant, reaching minimum values at the 30%PL composite. A similar effect was observed by Vidales-Barriguete et al. in their study with gypsum composites and the incorporation of plastic aggregates from low-voltage cables, where the decrease in open porosity and water absorption increased with the percentage of waste added, while thermal conductivity decreased [57]. This decrease in open porosity may be due to the “filler effect”, since the recycled PP/HDPE pellets occupy an increasing volume within the composite matrix, displacing part of the gypsum that would typically be in contact with the mixing water. This phenomenon reduces the space available for forming interconnected pores [62].

#### 3.2.2. Thermal Performance

**Figure 11:**
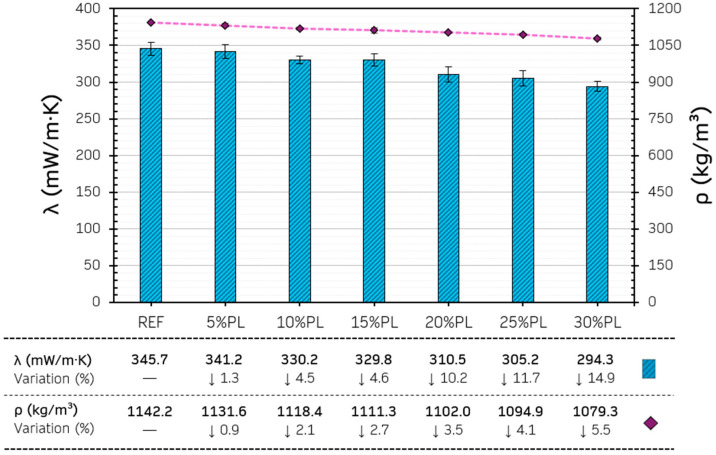
Results for density and thermal conductivity.

The thermal behaviour of gypsum composites is one of the key properties that must be analysed to assess their performance as an interior finishing material in buildings [63]. In this context, incorporating different additions, such as recycled plastic pellets, can improve these properties. Figure 11 presents the results obtained for the density and thermal conductivity of the gypsum-based composites with additives.

As can be seen in the results obtained in Figure 11, there was a progressive decrease in the composites’ thermal conductivity and bulk density as the content of recycled PP/HDPE pellets increased. Although this variation is slight, the 30%PL series showed a 5.5% reduction in bulk density compared to the reference (REF). This subtle variation may significantly affect the transport and logistical distribution of the final formed gypsum precast products, where the generation of economies of scale is key to increasing competitiveness in the market.

On the other hand, the variation in thermal conductivity was slightly higher, with a decrease of up to 14.9% from the reference in the composite with the highest pellet content (30% PL). These results show the positive effect of replacing the original gypsum content with these secondary raw materials. However, it is true that to obtain appreciable variations, a high replacement of the traditional material is required (in this case, 30% by volume). This variation in thermal conductivity cannot be attributed solely to the lower thermal conductivity of the recycled PP/HDPE pellets added. As highlighted in other research, incorporating these recycled raw materials into the composite matrix produces discontinuities, which can increase the overall porosity of the material and further reduce thermal conductivity [64]. The increased heterogeneity in the composite matrix caused by incorporating these pellets with a lower thermal conductivity coefficient is, according to several studies, the main factor to consider when analysing this phenomenon [8].

**Figure 12:**
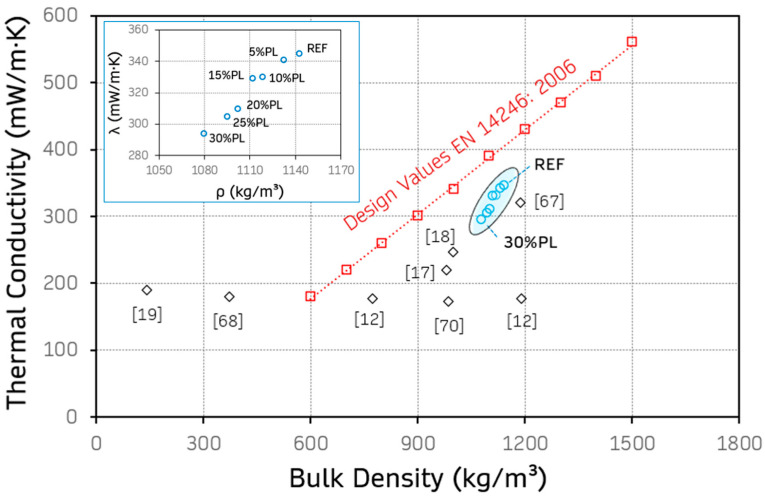
Discussion of thermal conductivity results, including experimentally obtained values (blue) and recommended values for precast design according to EN 14246:2006 [65]. References: [12] gypsum with 40% wt. of CD waste less than 4 mm; [66] gypsum with 3.5 wt. substitution by 1 mm cellulose acetate; [67] gypsum and recycled gypsum with 50% substitution of EPS less than 5 mm; [18] gypsum with 70% addition of cable waste less than 4 mm; [17] gypsum with 7.5% wt. substitution for single-use bag LDPE; [19] gypsum with 10% vol. substitution for HDPE; [68] gypsum with 30% vol. substitution for expanded polyethylene less than 3 mm.

For a more complete discussion of the experimental results obtained, Figure 12 shows the relative position of the gypsum-based composites developed compared to other studies in the literature. Additionally, the limit values of EN 14246:2007 were included to consider gypsum-based prefabricated products as lightweight and with low thermal conductivity [65].

Figure 12 shows that all the composites created in this research (shown in blue) were within the recommended values for use in prefabricated products, according to EN 14246:2006 [65]. However, compared to previous research, their density and thermal conductivity values were higher than those obtained for EPS-incorporated gypsum [67], plastic cable waste, or expanded polyethylene [68]. However, it should be noted that the addition or substitution percentages used in these investigations were higher, and the thermal conductivity and density of these materials also differed. These results agree with those presented in Figure 7, where higher mechanical strengths were exhibited for the gypsum-based composites developed compared to these same studies, where it was also noted that the water/gypsum ratio was a factor to be considered in this analysis [64].

#### 3.2.3. Simulation of Thermal Behaviour in Building Systems

**Figure 13:**
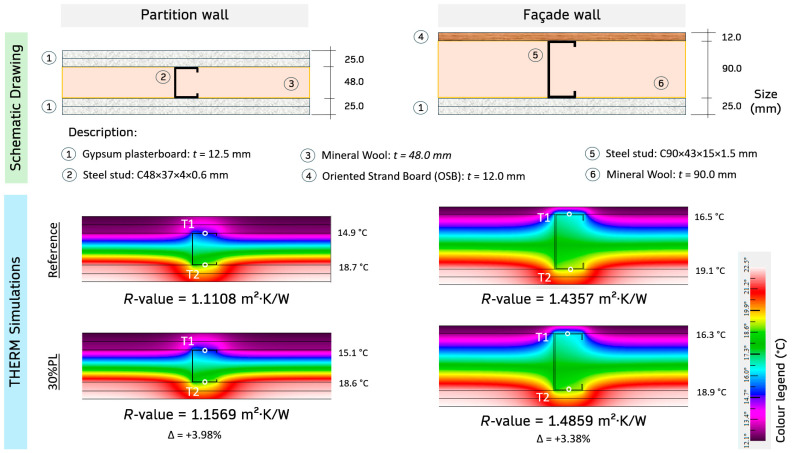
THERM simulation of thermal resistance for partition and façade walls using REF and 30%PL composites.

Table 6 presents the thermal conductivity and bulk density of the materials that compose the walls analysed. Figure 13 shows a schematic section and the finite element simulation results of the two proposed configurations with plasterboards based on the 30%PL composite and the reference. This simulation tool has been used in other studies as a verification method for experimental tests to determine the thermal behaviour of building systems [69].

Although the reduction in thermal conductivity of the gypsum composites produced was up to 14.9%, their contribution to overall wall performance is much smaller due to the reduced thickness of the plasterboards. The thermal resistance obtained for the partition, with a total wall thickness of 98 mm, was 1.1108 m^2^·K/W. By incorporating the panels made with the composite designed in this research, an increase in thermal resistance of 3.98% was obtained. The variation observed in the façade when using the panels with the new composite compared to the reference was slightly lower (3.38%). In the second type of wall, which has a thicker insulation layer, the panels’ effect on overall thermal resistance was more limited. It should be noted that the highest percentage of energy consumption is attributed to the thermal conditioning of spaces (up to 63.1% [74]), so increasing the thermal resistance of envelopes is essential for improving building energy efficiency and thus meeting the energy targets set by the European Commission for 2030.

### 3.3. Environmental Impact Analysis

**Figure 14:**
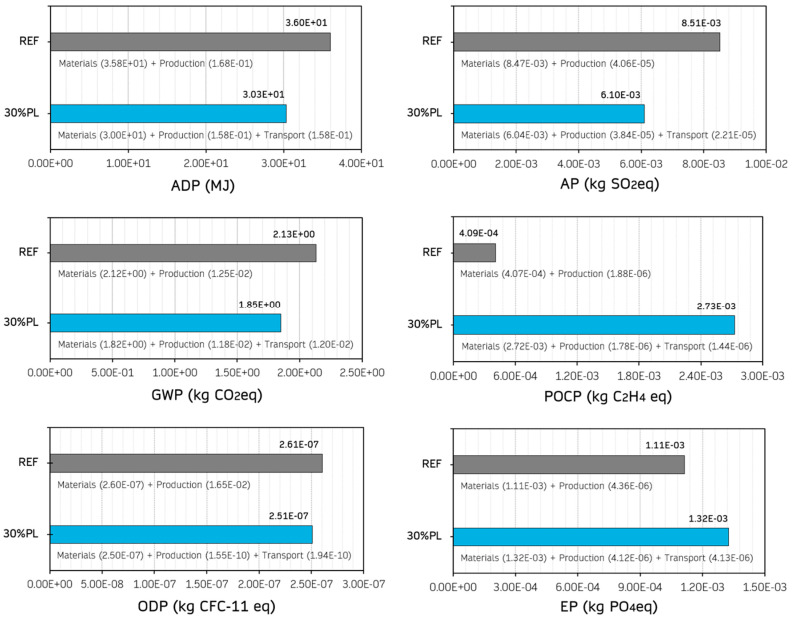
Environmental impact indicators determined for 1 m^2^ of prefabricated panel for the REF and 30%PL series. ADP, Abiotic Depletion Potential of Fossil Resources; AP, Acidification Potential; GWP, Global Warming Potential; POCP, Formation Potential of Tropospheric Ozone; ODP, Ozone Layer Depletion; and EP, Eutrophication Potential.

With about 21% of total global greenhouse gas emissions, the construction industry contributes strongly to climate change [75]. Developing new products under circular economy criteria is essential for promoting sustainable building. Figure 14 shows the results obtained for the life cycle analysis (LCA: cradle-to-gate) carried out for a 1 m^2^ prefabricated panel with the gypsum-based composites REF and 30%PL. Only these two gypsum-based composites were selected, since the recycled PP/HDPE pellet content was added progressively as a partial substitution for the traditional material, and a linear behaviour was inferred for the intermediate series.

Given the results presented in Figure 14, replacing traditional gypsum material with recycled PP/HDPE pellets led to a decrease in the impact on four of the six indicators analysed: ADP (↓15.8%), GWP (↓13.1%), ODP (↓3.8%), and AP (↓28.3%). However, incorporating these secondary raw materials increased the potential for photochemical ozone formation and eutrophication because of the emissions associated with the transport and production of these pellets. Although CO_2_ equivalent emissions are reduced, it is essential to consider all stages of the manufacturing process of these gypsum-based composites and their associated emissions in order to try to reduce the environmental impact of producing these prefabricated panels. A similar effect was observed in studies where 30% of the original gypsum material was replaced by expanded polyethylene [18]. However, these results were generally considered encouraging for improving the environmental performance of traditional gypsum-based composites.

At this point, it is necessary to make a final reflection on the implications of this research. Currently, plastics have become the most common type of marine litter, the generation of which is influenced by several factors. Firstly, economic factors are connected to the rising production of this waste as industrialisation increases [24]. On the other hand, social factors are linked to higher population density, the growth of urban infrastructures and coastlines [76], and environmental regulation policies associated with each geographic region [77]. In addition, a third component involves factors such as the wind patterns specific to each affected area [78]. The recovery, recycling, and revalorisation of marine litter have become a real and topical need in this multifactorial context, which contributes to plastic waste generation.

The construction sector plays a key role as part of the economic engine of nations and therefore has an added responsibility in its commitment to the environment. It is thus necessary to promote research, such as in this manuscript, to explore the recycling and reincorporation of different types of secondary raw materials of plastic origin deposited in the seas and oceans. At this point, gypsum, as a construction material, is widely used in Europe and especially in Mediterranean countries [79] and is postulated as an ideal matrix for integrating these by-products. This way, prefabricated plates and panels that lack structural character and have excellent versatility and vast potential for building applications can be generated.

## 4. Conclusions

In this research, a physico-mechanical characterisation of different gypsum composites with the incorporation of recycled PP/HDPE pellets was conducted. Plastic waste from recycling fishing nets was added—up to 30%—as a partial substitute for traditional materials. The most relevant conclusions that can be drawn from the analysis of the composites developed are as follows:

- All the composites analysed exceeded the minimum requirements for flexural (1 MPa) and compressive (2 MPa) strength as per EN 13279-2. Furthermore, the gypsum-based composites produced exhibited good mechanical behaviour compared to other gypsum materials with plastic waste reported in the literature. Even in the most unfavourable case (30%PL), the flexural (2.72 MPa) and compressive (7.15 MPa) strengths obtained are suitable for use in buildings. At the same time, SEM analysis showed the formation of CaSO_4_-2H_2_O crystals in the interphase skin matrix.
- The gradual incorporation of recycled PP/HDPE pellets in place of the original gypsum material enhanced water performance. In the most favourable case (30%PL), capillary water absorption was reduced by up to 20.5% compared to the reference. Similarly, a decrease in open porosity (↓9.3%) and total water absorption (↓8.8%) was observed relative to the reference series.
- This plastic waste caused a slight reduction in the density of the traditional material (↓5.5%) and in the thermal conductivity, which decreased to 292.3 mW/m·K (a 14.9% decrease). This reduction significantly enhanced the thermal resistance of lightweight cladding, increasing it by 3.98% in interior partitions and 3.38% in façades.
- In the cradle-to-gate LCA performed for the REF and 30%PL series, the composite with the highest recycled material content showed reductions in four of the six environmental indicators analysed: ADP (↓15.8%), GWP (↓13.1%), ODP (↓3.8%), and AP (↓28.3%). This represents a positive outcome in promoting the use of plastic waste for producing gypsum-based prefabricated products.

Overall, this work offers an alternative to traditional gypsum-based materials used in manufacturing prefabricated products and aims to incorporate circular economy principles into the development of these building materials. However, some limitations and future research directions should be noted. Firstly, it would be beneficial to investigate the fire behaviour of these composites, including their structural integrity and potential emissions during pellet combustion. Additionally, it would be valuable to examine the acoustic absorption coefficients of these innovative materials. Lastly, expanding the LCA to encompass the use and application phases of the evaluated prefabricated materials could be advantageous, as increased thermal resistance in enclosures may enhance the environmental performance of these composites.

## Figures and Tables

**Table 1:** Review of the current state of knowledge of research on gypsum-based composites with recycled plastic pellets.

Ref.	Plastic Type *	Size (mm)	Addition	Main Results
[12]	PC	Φ < 4.0	10–20–30–40% wt.	Waste from CDs and DVDs is used, making the composites lighter and reducing thermal conductivity by up to 29.2%. The composites integrate well into the matrix, although mechanical strength is reduced with additions of more than 10% wt.
[13]	PP	Φ < 4.0	2.5–5.0–7.5–10.0% wt.	Pellets from discarded coffee capsules show a decrease in flexural strength, but compressive strength is not affected. There is an improvement in resistance to water action.
[14]	Mix	Φ < 1.0 1.0 < Φ < 1.3 1.6 < Φ < 3.0	5.0–10.0–15.0% wt.	The progressive increase of recycled plastic aggregates significantly improves thermal resistance. However, gypsum’s workability is reduced, and its mechanical properties are adversely affected.
[15]	EPS	Φ = 5.0	1.0% wt.	A gypsum material with high thermal resistance is developed for 3D printing. Its mechanical properties are strongly affected, limiting its field of application.
[16]	PUR	Φ < 2.0	3.3% wt.	Prefabricated boards with a lower environmental impact are being developed. For the commercial gypsum product, a decrease of 14% in energy consumption, 14% in CO_2_ emissions, and 25% in water demand is estimated.
[17]	LDPE	Φ < 4.0	2.5–5.0–7.5% wt.	Plastics recovered from single-use bags. The density of the composite is reduced, its performance against water is improved, and its thermal conductivity is reduced, while its mechanical properties are negatively affected.
[18]	Plastic cable	Φ < 3.0	50.0–60.0–70.0% wt.	Prefabricated plates with a high content of this recycled material are developed. Compared to the original material, the results show a reduction in flexural strength and a decrease in thermal conductivity.
[19]	HDPE	Φ = 1.4	2.0–4.0–6.0–8.0–10.0% vol.	The total water absorption and thermal conductivity of the gypsum composites are significantly reduced. However, there is a reduction in mechanical strength and weak integration of the residue in the matrix.

* PC = Polycarbonate; PP = Polypropylene; EPS = Expanded Polystyrene; PUR = Polyurethane; LDPE = Low-Density Polyethylene; HDPE = High-Density Polyethylene.

**Table 2:** E-35 gypsum properties as specified by the manufacturer Saint-Gobain Placo Ibérica, S.A. [34].

**Granulometry (mm)**	0–0.2	**Vapour diffusion factor (µ)**	6
**Fire reaction**	A1	**Thermal conductivity (W/m·K)**	0.30
**Flexural strength (MPa)**	>3.0	**pH**	>6

**Table 3:** Recycled PP/HDPE materials properties [35].

**Real density (kg/m^3^)**	0.923 ± 0.001	**Tensile strength deformation (%)**	9.60 ± 0.10
**Impact strength (kJ/m^2^)**	3.26 ± 0.06	**Flexural strength (MPa)**	37.10 ± 0.11
**Tensile strength (MPa)**	28.40 ± 0.55	**Flexural strength deformation (%)**	7.60 ± 0.07

**Table 4:** Mix proportions employed in the sample preparation.

Material	REF ^1^	5%PL ^2^	10%PL	15%PL	20%PL	25%PL	30%PL
Gypsum (g)	1000.0	950.0	900.0	850.0	800.0	750.0	700.0
Water (g)	650.0	617.5	585.0	552.5	520.0	487.5	455.0
PP/HDPE (g)	—	23.3	46.7	70.0	93.3	116.7	140.0

^1^ REF—Reference; ^2^ PL—Pellets.

**Table 5:** Inventory of compounds considered in the LCA.

Composites	Raw Materials (kg)			Transport (km)
	Gypsum	Water	Recycled PP/HDPE Pellets	
REF	16.28	10.58	-	-
30%PL	11.39	7.41	2.28	34

**Table 6:** Properties (thermal conductivity and bulk density) of the materials utilised in the simulation.

Material	Reference	λ (W/m·K)	ρ (kg/m^3^)
Gypsum plasterboard (Reference)	This study	345.7	1142.2
Gypsum plasterboard (30%PL)	This study	294.3	1079.3
Mineral wool (Thickness: Partition = 48.0 mm and Façade = 90 mm)	[70]	0.033	70.0
Oriented strand board (OSB)	[71]	0.130	600.0
Steel studs: C90 × 43 × 15 × 1.5 mm and C48 × 37 × 4 × 0.6 mm	[72,73]	50.000	≈7850.0

## Data Availability

The original contributions presented in this study are included in the article. Further inquiries can be directed to the corresponding author.
